# Balancing Assessment with “In-Service Practical Training”: A Case Report on Collaborative Curriculum Design for Delivery in the Practice Setting

**DOI:** 10.3390/pharmacy7030093

**Published:** 2019-07-16

**Authors:** Cicely Roche, Michelle Flood, Matthew Lynch, Laura J. Sahm

**Affiliations:** 1 School of Pharmacy & Pharmaceutical Sciences, Trinity College, Panoz Building, College Green, Dublin 2, Ireland; 2 School of Pharmacy, Royal College of Surgeons, Dublin 2, Ireland; 3 Pharmaceutical Care Research Group, School of Pharmacy, University College Cork, Cork T12 YN60, Ireland; 4 Department of Pharmacy, Mercy University Hospital, Grenville Place, Cork T12 WE28, Ireland

**Keywords:** in-service practical training, peer-learning, rubrics, integrated curriculum, co-developed modules, online learning

## Abstract

Three Higher Education Institutions (HEIs) in Ireland are accredited to provide education and training, successful completion of which, entitles one to register as a pharmacist with the Pharmaceutical Society of Ireland (PSI). Legislation (2014) mandated that these HEIs replace their existing structure (four-year degree followed by a one-year internship), with a five-year ‘integrated Master’s programme’. Integration includes ‘in-service practical training’ (placement) at the beginning of Year 4 (four months), and the end of Year 5 (eight months). Year 4 placements do not have to be ‘patient-facing’. Students receive a Bachelor’s degree at the end of Year 4. The Affiliation for Pharmacy Practice Experiential Learning (APPEL), established by the HEIs, manages student placements, training establishments, preceptor training, the preceptors’ competency assessment process, and the virtual learning environment (VLE) that enables delivery of co-developed online modules aligned with placements in Years 4 and 5. This case report aims to describe the process by which this integration has taken place across and within these HEIs and the challenges faced by educators, students, preceptors, and other stakeholders along the way.

## 1. Background and Introduction

In Ireland, three Higher Education Institutions (HEIs)—the Royal College of Surgeons in Ireland (RCSI), Trinity College Dublin (TCD), and University College Cork (UCC)—are accredited to provide programmes of ‘education and training’, graduates of which are entitled to apply to register as a pharmacist [1]. Prior to September 2015, students completed a four-year undergraduate degree, followed by a 12-month period of pre-registration training: The National Pharmacy Internship Programme (NPIP) [2]. A review of undergraduate and pre-registration training, the Pharmacy Education and Accreditation Review (PEARs) project [3] was commissioned by the Pharmaceutical Society of Ireland (PSI) and recommended the introduction of an integrated five-year Master’s level programme of education and training for pharmacists. The PSI accepted the PEARs findings and following legislative change in 2014, the five-year integrated programme was introduced in the three HEIs in September 2015 [4]. The PSI’s Core Competency Framework (CCF) for Pharmacists [5] was a mandatory component informing the design of the integrated programmes in each HEI. The CCF delineates 6 domains of practice, and associated 25 competencies. The CCF informs the PSI’s standards for accrediting pharmacy degree programmes [5,6]. Curriculum design must therefore map to the CCF. The reference to ‘integrated’ includes ‘in service practical training’ (placement) for four months at the beginning of Year 4, and for the final eight months of Year 5 [6]. An overview of the CCF is provided in Table 1.

*Aim and Objectives*

The aim of this case report is to detail the process by which HEIs in Ireland collaborated to design a common curriculum, integrated both within and across HEIs, for delivery to students while undertaking placement in Year 4 and to consider the associated challenges faced by educators, students, preceptors, and other stakeholders.

The objectives are to describe this process according to three distinct constituent phases as follows: planning, implementation, and review.

*Planning Phase:*

(i) To describe the structures necessary to operationalize these new requirements.
(ii) To explain the alignment of student contact hours in the online modules with placement hours.
(iii) To define Preceptors and their scope.
(iv) To outline the criteria for curriculum design.
(v) To define and explain the two stages of the work programme, i.e., Stage 1: Establishment of Working Group (WG) and Stage 2: Curriculum Design.

*Implementation Phase:*

(i) To designate the outcomes achieved and their linkage to CCF, learning outcomes, assessment, teaching, and learning.
(ii) To describe the outputs achieved, and to discuss in the context of the literature.

*Review Phase:*

To describe the challenges, learnings, and recommendations for future iterations

## 2. Planning Phase

### 2.1. Structures

The three HEIs established the Affiliation for Pharmacy Practice Experiential Learning (APPEL) with the Head of each HEI being appointed to its Board of Directors. APPEL is responsible for the management of (a) student placements; (b) training establishments; (c) preceptor-student matching; (d) preceptor training; and (e) the workplace-based competency assessment process [6]. It also manages the virtual learning environment (VLE) that enables delivery of co-developed online modules aligned with placement in Years 4 and 5.

With respect to training establishments, unlike in Year 5 where placements are restricted to hospital or community settings, students in Year 4 also have a range of non-clinical options available for placements—e.g., in a pharmaceutical company, a pharmaceutical wholesaler, or a regulatory body [6]. Additionally, in some circumstances the student may undertake a Year 4 placement outside of the State [7]. As many placement establishments are geographically removed from the HEIs, students would not be able to attend the HEI while on placement, therefore all activities aligned with the online modules, including assessment, needed to be completed at a distance on the APPEL VLE.

As Year 4 placement options do not necessarily facilitate students engaging directly with patients, curriculum design must preclude any activity or assignment that requires student access to patients or patient-records. Accordingly, only three of the six domains in the CCF, namely: professional practice, personal skills, and organisation and management skills (Table 1), are directly aligned with the online modules for Year 4.

### 2.2. Alignment of Student Contact Hours

Degree awards in HEIs in the Republic of Ireland align with the European Credit Transfer and Accumulation System (ECTS). These credits represent the workload and defined learning outcomes in a programme [8]. Sixty ECTS, representing 1500–1800 h of student effort, are equivalent to a full year of study or work in an undergraduate degree programme [8]. As the Year 4 placement aligns with half the academic year for an undergraduate student, this represents 30 ECTS of placement time and study, and the corresponding student workload in each HEI is set at a maximum of 750 h (equivalent to 50 h per week for 15 weeks). The Board of APPEL agreed that students would complete 30 h per week in their placement, between the hours of 9 a.m. to 5.30 p.m., four days per week, during Monday to Friday. Therefore, curriculum design had to take account that the time remaining after that assigned to placement activities that could be allotted to Year 4 online modules was a maximum of 20 h per week.

### 2.3. Preceptors and Their Scope

Preceptors are registered pharmacists who agree to mentor and assess students on the skills that they demonstrate. Their role (in Year 4), is to complete the competency assessment of the student and determine whether, upon completion of the placement, the student has achieved a minimum of, a Level 3 (“mostly”) rating (Table 2) in all relevant behaviours (51 behaviours) (see Appendix A, Table A1).

While the competency assessment has no marks associated with it per se, it is a compulsory element that must be successfully completed—i.e., attain a Level 3 award in the designated competencies—in order to pass the module. Further discussion on the competency assessment process is outside the scope of this case report.

### 2.4. Criteria for Curriculum Design

**Figure 1:**
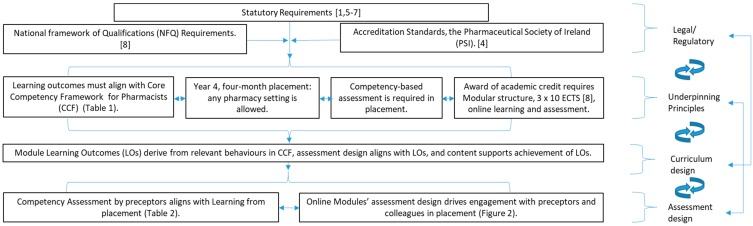
Interrelationships between legal requirements, underpinning principles, and curriculum and assessment design.

When designing the curriculum for students completing placements in Year 4 of the programme, it was necessary to take account of a complex series of interrelationships, summarised in Figure 1.

APPEL provided further requirements that further informed the curriculum design as follows:

- Materials should be co-delivered to students from all three HEIs while on Year 4 placement, from September to December of that academic year;
- there should be no requirement for students to attend the HEI at any stage during the placement;
- activities that require access to patients or patients’ records should be excluded;
- learning outcomes to be derived from the same CCF behaviours as used for the placement assessment;
- alignment with 30 ECTS academic credit for Year 4 of the Degree award, and with student workload of a maximum 20 h per week for a total of 15 weeks is a requirement;
- there should be no additional preceptor workload when designing activities/assessment; and
- standards and/or regulations for curriculum design, and for progression and award of Degrees, at all three HEIs were to be accommodated.

### 2.5. Stages of Work Programme

Stage 1: Establishment of Working Group

APPEL appointed a working group (WG), composed of one pharmacist nominee from each of the HEIs (CR, MF, and LJS) charged with drafting a proposal by December 2017 for a curriculum design that would be common to all HEIs and which would be co-delivered to students while undertaking practice placements in Year 4. APPEL stipulated that the curriculum design should have a University character, including its expectation that there would be synchronous, online activity on a regular basis—preferably no less than once weekly.

All members of the WG had experience in curriculum design, online learning, and assessment. A Practice educator at RCSI/APPEL, the project manager at APPEL, and the instructional designer at TCD provided support to the WG, and each member of the WG collaborated with colleagues in their respective HEIs, working towards the preparation and delivery of curriculum materials in September-December 2018.

Stage 2: Curriculum Design—Alignment of Learning Outcomes with Activities and Assessment

The WG adopted a student-centred, outcomes-focused approach to curriculum design [8,9,10] and prioritised integration of content and activities to the assessment design [11,12,13]. Rubrics were sufficiently detailed to guide both students and assessors [14]. Online modules were designed to be in synergy with the experiential nature of placements—i.e., to support learning by doing while on placement [15,16,17]. Developmental approaches to reasoning and decision-making through the ambiguity experienced in ‘practice’ [18,19,20], as previously used in online and blended learning in Pharmacy education in Ireland [21,22,23], were a central component of curriculum design.

The WG committed to providing a guided approach to reflective writing [24,25] and, combined with promoting repeated cycles of reflection [15,16,26], this commitment further informed the format, sequencing, and timing of activities and student submissions [27,28,29]. Individual and social constructivist theories [15,30], which propose that learning is an active process wherein new information is added to ‘prior knowledge’ which may have been derived from experience as well as formal teaching and learning, and that can take place on an individual or social basis, underpinned curriculum design. Assessment that was simultaneously ‘of’ (demonstrating achievement), ‘for’ (to provide feedback by e.g., self and peer assessment against comparators), and ‘as’ (wherein students self-regulate) learning [31,32,33] was the aim. Peer interaction and debate [34,35] was stimulated by appropriate sequencing of the order in which students completed activities [20,21,22,23]. Selection of team tasks, and Community of Inquiry methodologies which prompted emphasis on social presence (e.g., interacting in teams to agree decisions), cognitive presence (e.g., activities requiring critical and independent thinking), and teaching presence (i.e., the establishment of a defined process to drive reflection and interaction online) [36] further encouraged student engagement [37,38] with their online groups, and with the range of experts (e.g., preceptors) accessible during their placements. Participation in peer review and feedback [39], required for programme accreditation purposes [3], was introduced as a group activity. The WG aimed to maximise the potential arising from an entirely online learning, feedback and assessment environment [40], especially with respect to demonstration of professionalism and online etiquette (“netiquette”) when interacting with peers [41].

The WG convened on six occasions for two days meetings, from April to October 2017, and issued progress reports, including recommendations for decision to the Board of APPEL, after each meeting.

**Figure 2:**
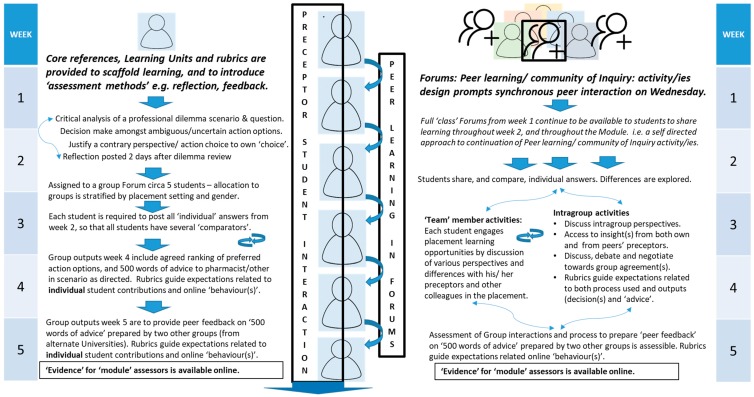
Student learning process aligned with online modules and Assessment *of*, *for* and *as* learning. * Assessment *of (to demonstrate achievement), for (to provide feedback by e.g., self or peer assessment against comparators)* and *as* learning wherein students self-regulate) [31].

An overview of the assessment design, and its alignment with content, activities and the placement, is provided in Figure 2.

## 3. Implementation Phase

The curriculum design for modules for Year 4 were progressed in terms of:

### 3.1. Module Links to CCF

- The WG reviewed behaviours listed in the three Domains in the CCF and recommended that 51 behaviours be included (Appendix A, Table A1).
- Each module was linked to a number of CCF competencies and behaviours relevant to the range of placement types possible in Year 4 (Appendix A, Table A1).
- These behaviours guided the development of LOs for each module.

### 3.2. Module Learning Outcomes (LOs)

- Module learning outcomes were devised based on covering the cognitive (knowledge, comprehension, application, analysis, synthesis), affective, and psychomotor domains relevant to a student in Ireland. [42]
- Eight LOs were agreed for each module, of which three LOs were common across each module—i.e., (i) Participate in accordance with the behaviours in domain [x] of the CCF; (ii) Integrate knowledge and skills to ensure safe and effective practice; and (iii) Demonstrate engagement in reflective practice and continuing professional development.

### 3.3. Approaches to Assessment

- Each module included one case-based assessment, to assist in achieving the LOs, that requires (i) Individual and group work, and (ii) self- and peer assessment (Figure 2).

### 3.4. Approaches to Teaching and Learning

- Student workload was determined to be ten hours per week on structured learning (directed learning (DL)) activities, and 10 h per week on personal learning (self-directed learning (SDL)) activities.
- 30 h per week were assigned to placement related activity.
- Reference to DL included provision of nine 20 min interactive vodcasts/Learning Units (LUs) and two ‘core references’, that collectively supported achievement of the LOs and completion of assignments in each module.

An outline module descriptor for the first module delivered is provided in Appendix A, Table A2.

### 3.5. Outputs

- Three 10 ECTS Modules, each of which aligned with 200 to 250 h of student effort, [43] were developed.
- Credits aligned with 10ECTS are divided between individual (50%) and group activities (50%). Nine LUs were developed, and weekly synchronous, online activities which include self-directed learning, individual and group activities are aligned with scheduled time online, between 1:00 p.m. and 5:00 p.m. on Wednesday afternoons.
- Case video (Appendix A, Table A3) is used as a vehicle to pose relevant questions and motivate discussion within groups. Two core references, available to students prior to viewing the video, provide background information for the case.
- All student activities/outputs were collated to the group’s online discussion Forum i.e., all ‘evidence’ is available in the one discussion Forum.
- Detailed rubrics, with a total of 10 criteria, provided guidance to students and to assessors. (See Appendix A, Table A4 for individual activities rubric and Appendix A, Table A5 for group activities rubric).
- A student guidance booklet and a module co-ordinator information booklet were developed.
- Weekly announcements, reminding students of activities and related expectations of them during that week, were prepared.
- A weekly schedule, of student learning and assessment, is provided in Appendix A, Table A6.

The module was delivered to students from all three HEIs on time, and all activities were completed as required. LUs and core references were accessed by all students, averages grades were comparable across the HEIs, and were in line with what would be expected at Year 4 of the Bachelor’s Degree. The range of marks awarded to students was acceptably in line with institutional norms.

## 4. Review Phase

Following the implementation phase, a review of the initial iteration of the Year 4 programme was undertaken. Arising from this, various challenges, learnings, and recommendations emerged as follows.

### 4.1. Challenges

The technology raised challenges regarding risks of plagiarism and impersonation. While efforts to manage this risk included the use of detailed rubrics, the expectation that students make multiple contributions to module discussion forums and that rubrics allow the alignment of grades to meaningful contributions in group discussions, there remained some risk of online impersonation and/or plagiarism.

Incorporation of online groups included the need for assurance that activities occur in a timely fashion. Accordingly, the design process must pay particular attention to guidelines that prompt timely engagement by all group members, so that those engaging in the early stages do not become despondent with the online teamwork.

Allocation of 50% of module marks to the individual component, which restricted students to a 500 words submission, required students to accommodate a significant change in their usual approach to such assignments. This limited word count aligned with the experiential nature of placements, wherein patient notes and recordings of events must be complete yet cogent and concise.

The requirement that students had to be online synchronously on Wednesdays was queried by various parties including preceptors wanting students to engage in other placement activities occurring at that time. However, the realization that one late or non-engaging student can impact on their entire group’s performance, combined with the stipulation by APPEL that there be must be regular synchronous online activity, generally overcame this difficulty.

### 4.2. Learnings

Concerns raised by academic colleagues across each HEI, that there would be no summative examination and that group work could lead to grade inflation and/or poor grade differentiation, did not materialise as the mean and distribution of grades across each module were within institutional norms.

Rubrics, collaboratively prepared by the module leads in each HEI, motivated detailed planning regarding grading expectations and supported consistency in approach to subsequent module development—i.e., the rubric format remained constant across modules—and serendipitously much of the rubric descriptors required little amendment for subsequent modules (Appendix A, Table A4 and Table A5).

Assessment load for assessors for the first module is estimated at 30 to 40 min per student. This estimation includes two face-to-face meetings involving the module leads from each HEI for the purpose of quality assurance within the module whereby the leads collectively reviewed a sample of individual and group work, prior to grading and discussed the approach to grading. Notwithstanding that streamlining of the administrative elements may reduce time required in future cycles, this time allocation is considered to be acceptable for a 10 ECTS module in Year 4.

While the use of a shared VLE whose management was outsourced externally required advance agreement across the three HEIs regarding content and process, and restricted the freedom of leads to change material, this ensured a common template and structure for students across each HEI.

### 4.3. Recommendations for Future Work

Staff development should be prioritized in a number of areas including (i) moderating and grading online forums, (ii) development of rubrics, and (iii) development of reflective writing skills in students.

The collaborative approach of the working group should be explored as a potential framework for development of cross-institutional curriculums.

## 5. Discussion and Conclusions

The aim of this case report was to detail the process by which HEIs in Ireland collaborated to design a common curriculum, integrated both within and across HEIs, for delivery to students while undertaking placements in Year 4 and to consider the associated challenges faced by educators, students, preceptors, and other stakeholders.

While the curriculum design process was informed by a number of important interrelationships (Figure 1) and APPEL requirements, the need for it to be student-centered and outcomes-focused [8,9,10], both from experiential learning perspectives (learning by doing while on placement [15,16,17]), and with respect to activities and assessment were key determinants. The student view was sought during development, when students attended the face-to-face pre-placement day and during delivery of the first module. The approach was organic in that changes were made as required to take account of particular situations that presented for either individual students or the wider student cohort—e.g., when students requested a later submission time for one assignment to accommodate commuting time from their training establishments. Themes for case scenarios in the three online modules (unlicensed medicines usage, interpersonal relationships and pharmacoeconomic assessment) reflected issues relevant to the profession as a whole, thereby exposing students to a range of dilemmas typically faced in pharmacy practice. As allocation to groups was stratified by training establishment and by gender, groupwork enabled learning from (i) peers in different practice contexts, (ii) collective engagement with a range of preceptors in their role as ‘experts’, and (iii) interaction with others in the training establishment [15,16,17] and Figure 2. Constructivism, both individual and social, is the key learning theory used [15,26,27,31,32,33]. A guided approach to reflective writing was provided, using both LUs and the rubric to directly support students’ ability to engage with the four levels of written reflection [24,25]. By revisiting their own initial response, each student was encouraged to reflect on learning from ‘practice’. Group work activity facilitated further expansion of the range of perspectives that could support resolution of the dilemma(s) presented in the relevant scenario.

Integration of learning with the assessment design was achieved by designing activities to act as catalysts for learning from module content and from interaction with the placement, and by setting up and managing the learning environment in a manner that supported students through activities in a specific sequence (Appendix A, Table A6) [11,12,13]. Student completion of activities provided evidence of ‘achievement’ that could be objectively assessed. Design of assessment that is ‘*of*’, ‘*for*’, and ‘*as*’ learning reduces assessment load for students [31]. Detailed rubrics were provided to students at the beginning of the module thereby clarifying expectations and supporting learning [14]. When assessing student work, each module lead ‘benchmarked’ submissions against the rubric, thereby enabling student self-assessment prior to engaging in assignments for subsequent modules [39].

Developmental approaches to reasoning and decision-making [18,19,20,21,22,23]—namely the incorporation of logic or relevant decision-making frameworks, role-play, and peer interaction [18,34,35]—were a central component of the curriculum design. The LUs added to the knowledge accrued in Years 1–3 of the programme. These also emphasised decision-making frameworks (Appendix A, Table A2), and the requirement that each student, first independently, identify ethical concepts in the dilemma and justify what action should be taken, introduced critical and independent thinking/individual constructivism, logic, and role-play to the process (Appendix A, Table A3 and Table A6) [35]. Role-play, or the taking account of the perspectives of others, was achieved when students (i) individually completed the ranking of ‘less than ideal’ action options and (ii) explained how a pharmacist might try to justify the action options that the student chose as least preferred.

Having committed to individual choices regarding rating and ranking of action options offered, students had to agree a group decision regarding the ranking process within a defined time-frame (Figure 2). This inevitably involved negotiation and active discussion, debate and persuasion as the group sought to complete the task by the deadline imposed. The debate also demanded deeper reflection on the student’s individual decisions made prior to discussion with their peers. Peer interaction was stimulated by appropriate sequencing of the order in which students completed activities [20,21,22,23,34,35]. Group composition was changed for subsequent modules, thereby forcing repeated exposure to ‘new’ perspectives and approaches to decision-making.

The design enabled and stimulated students to participate as online ‘Communities of Inquiry’ while collectively sharing the expertise available from preceptors practising in a range of contexts. The requirement to collaboratively prepare 500 words of advice promoted and facilitated student engagement [36,37,38] with the range of experts (e.g., preceptors) accessible during their placements. The provision of peer feedback on ‘advice’ prepared by groups from other HEIs required each member to engage with perspectives of their ‘own’ peers who had studied in different contexts, while also developing the ability to review and feedback on colleagues’ work. Students become familiar with the learning and assessment process in this first module and, by repeating the process in subsequent modules, students have opportunity to adapt this ‘expertise’ so that decision-making through ambiguity becomes tacit, or acquired at a ‘bedrock’ level [18,19,20,21,22,23].

The WG used the potential arising from the online learning environment [39] for additional benefit such as to (i) identify whether students had successfully engaged with all online activity ‘types’ during orientation week and provide struggling students with support prior to the start of the module; (ii) identify that all students completed required individual assignments during the defined timeframe(s), especially during week 2; (iii) provide evidence of, or lack thereof, professionalism in forums; and (iv) accommodate different learner styles by means of incorporating both synchronous and asynchronous interaction [40]. Benchmarking against a detailed rubric enabled feedback from module leads in a timely fashion, while rubric descriptors for higher levels than achieved by students (Appendix A, Table A4 and Table A5) provided guidance to students as they self-assessed how they might improve performance in subsequent modules.

As all contributions were recorded online, they collectively provided evidence of the standard to which students had engaged in the process in a format that accommodated individual HEI rules and regulations related to examinations, and decisions related to student progression and awards.

In Section 4, the outcome from the review undertaken after the first iteration of the programme was detailed.

In conclusion, while the overall rollout was successful, it was not without its challenges and there are important learnings that will be taken on board both for future iterations of Year 4 and for the delivery of an analogous education programme for Year 5. Furthermore, they offer valuable insights for those who may be contemplating the development of similar programmes to be delivered in the practice setting in a related educational context.

## Figures and Tables

**Table 1:** Core Competency Framework (CCF) for Pharmacists.

Domain	Competency
Professional practice	Practises ‘patient-centred’ care Practises professionally Practises legally Practises ethically Engages in appropriate continuing professional development
Personal skills	Leadership skills Decision making skills Team working skills Communication skills
Supply of medicines	Manufactures and compounds medicines Manages the medicines supply chain Reviews and dispenses medicines accurately
Safe and rational use of medicines	Patient consultation skills Patient counselling skills Reviews and manages patient medicines Identifies and manages medication safety issues Provides medicines information and education
Public health	Population health Health promotion Research skills
Organisation and management skills	Self-management skills Workplace management skills Human resources management skills Financial management skills Quality assurance

(PSI, 2013:10) [5].

**Table 2:** Assessment Ratings.

Level	Rating	Definition
N/A	Cannot	Student not exposed to this behaviour in the training establishment.
1	Rarely	Very rarely meets the standard expected. No logical thought process appears to apply.
2	Sometimes	Rarely meets the standard expected. Much more haphazard than “mostly”.
3	Mostly	Standard practice usually met with occasional lapses.
4	Consistently	Demonstrates the expected standard practice with rare lapses.

(CoDEG, 2007) ([9] (adapted)).

**Figure A1:**
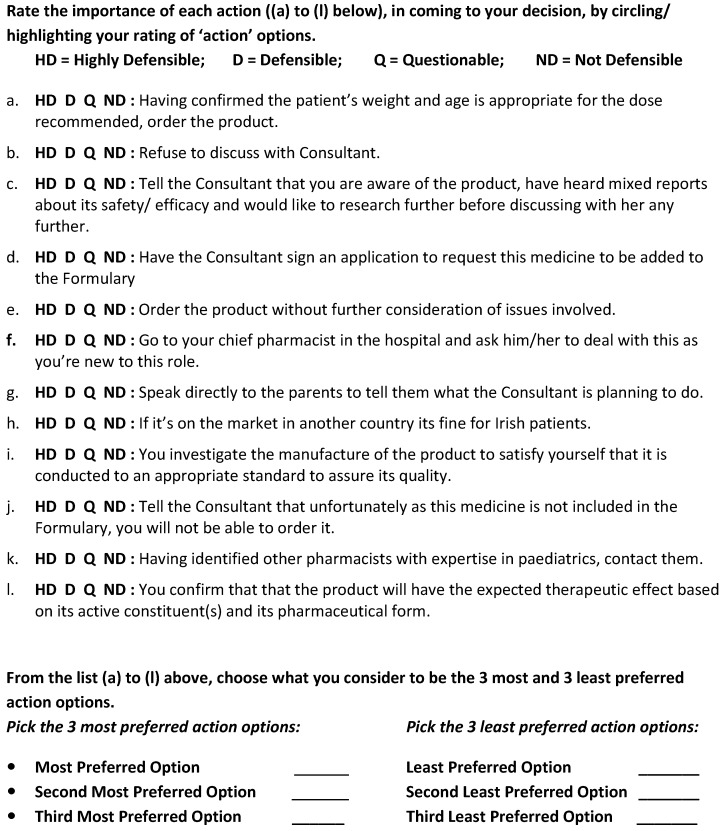
Range of 12 action options, related to the Case scenario (Box A1), provided to students.

## Appendix A

**Table A1:** CCF Behaviours aligned with Year 4 online modules [5].

CCF Behaviours Aligned with Year 4 Online Modules
**Professional Practice Module**
1.1 Practices ‘patient-centred’ care
1.1.1 Demonstrates a ‘patient-centred’ approach to practice
1.1.2 Ensures patient safety and quality are at the centre of the pharmacy practice
1.2 Practices professionally
1.2.2 Demonstrates awareness of the position of trust in which the profession is held and practises in a manner that upholds that trust
1.2.3 Treats others with sensitivity, empathy, respect and dignity
1.2.4 Takes responsibility for their own actions and for patient care
1.2.7 Recognises their scope of practice and the extent of their current competency and expertise and works accordingly
1.2.8 Maintains a consistently high standard of work
1.3 Practices Legally
1.3.2 Understands and applies the requirements of both Irish and European pharmacy and medicines law
1.3.3 Demonstrates an awareness of other legislation relevant to their practice setting including as appropriate data protection law, health and safety law, employment law, consumer law, equality law and intellectual property rights
1.3.4 Demonstrates an understanding of the requirements of the regulatory framework to authorise a medicinal product including the quality, safety and efficacy requirements
1.4 Practices ethically
1.4.1 Understands their obligations under the principles of the statutory Code of Conduct for Pharmacists and acts accordingly
1.4.2 Makes and justifies decisions in a manner that reflects the statutory Code of Conduct for pharmacists and pharmacy and medicines law
1.4.3 Recognises ethical dilemmas in practice scenarios and reasons through dilemmas in a structured manner
1.5 Engages in appropriate continuing professional development (CPD)
1.5.1 Understands and accepts the importance of life-long learning for pharmacists
1.5.2 Demonstrates the ability to critically reflect on their own practice and skills, to identify learning and development needs
1.5.3 Takes personal responsibility for engaging in CPD and achieving learning and professional development goals
1.5.4 Identifies and undertakes appropriate learning activities and programmes that meet identified learning needs
**Personal Skills Module**
2.1 Leadership skills
2.1.1 Inspires confidence and applies assertiveness skills as appropriate
2.1.2 Leads by example by acting to ensure patient safety and quality within the pharmacy environment
2.1.3 Builds credibility and portrays the profession in a positive light by being professional and well informed
2.2 Decision-making skills
2.2.2 Makes decisions and solves problems in a timely manner
2.2.3 Gathers information from a number of reliable sources and people to enable them to make well-founded decisions
2.2.4 Communicates decisions comprehensively including the rationale behind decisions
2.2.5 Ensures that relevant professional, ethical and patient safety factors are fully considered in decisions into which they have an input
2.2.6 Distinguishes between important and unimportant issues
2.2.7 Demonstrates an attention to detail and accuracy in decision-making
2.2.8 Recognises when it is appropriate to seek advice from experienced colleagues, refer decisions to a higher level of authority or to include other colleagues in the decision
2.3 Team working skills
2.3.1 Recognises the value and structure of the pharmacy team and of a multiprofessional team
2.3.5 Demonstrates a broad understanding of the services delivered by other healthcare professionals and disciplines
2.4 Communication skills
2.4.1 Uses effective verbal, non-verbal, listening and written communication skills to communicate clearly, precisely and appropriately
2.4.3 Uses appropriate language and checks understanding
2.4.4 Demonstrates respect, cultural awareness, sensitivity and empathy when communicating
2.4.5 Demonstrates influencing and negotiation skills to resolve conflicts and problems
**Organisation and Management Skills Module**
6.1 Self-management skills
6.1.1 Demonstrates organisation and efficiency in carrying out their work
6.1.2 Ensures their work time and processes are appropriately planned and managed
6.1.3 Demonstrates the ability to prioritise work appropriately
6.1.4 Takes responsibility as appropriate in the workplace
6.1.5 Demonstrates awareness of the responsibility of their position
6.1.6 Ensures punctuality and reliability
6.1.7 Reflects on and demonstrates learning from critical incidents
6.2 Workplace management skills
6.2.1 Demonstrates an understanding of the principles of organisation and management
6.2.2 Works effectively with the documented procedures and policies within the workplace
6.2.3 Understands their role in the organisational structure and works effectively within the management structure of the organisation
6.2.5 Addresses and manages day to day management issues as required in their position of responsibility
6.3 Human resources management skills
6.3.3 Engages with systems and procedures for performance management
6.3.4 Supports and contributes to staff training and continuing professional development
6.5 Quality assurance
6.5.1 Recognises quality as a core principle of medicines management and healthcare provision
6.5.2 Understands the role of policies and procedures in the organisational structure and in the provision of healthcare
6.5.3 Contributes to the development, implementation, maintenance and training of staff on standard operating procedures, as appropriate to their level of responsibility
6.5.4 Contributes to regular audit activities and reports and acts upon findings

**Table A2:** Module descriptor extracts Professional practice.

Module Title	Professional Practice (Extracts from Module Descriptor)
**Credit**	10 ECTS
**Elective/Mandatory**	Mandatory
**Sequence**	Year 4 Placement Module 1
**Indicative Weekly Schedule**	Directed/Structured 15 h placement activities 10 h online learning Self-Directed/Unstructured 15 h placement activities 10 h online learning
**Duration**	6 weeks [1 week orientation + 5 weeks of module]
**Module Coordinators**	Assoc. Prof. Cicely Roche (TCD) Dr. Laura Sahm (UCC) Dr. Matthew Lynch (RCSI)
**Pre-requisites**	Completion of year 3
**Module Rationale**	This module focuses on CCF Domain 1, Professional Practice and helps students develop the concept of what it means to be a pharmacist. The module will encompass the legal, ethical, and professional challenges faced by pharmacists in their working environment. It will also help raise awareness of the importance and necessity of lifelong learning.
**Module Aim**	This module aims to help students develop their knowledge, skills, and attributes in CCF Domain 1, Professional Practice.
**Learning Outcomes**	Participate in accordance with the behaviours identified in Domain 1 of the CCF Explain the legal requirements regulating the practice of pharmacy Integrate knowledge and skills to ensure safe and effective practice Critically review professional dilemma(s) Rationalise professional decision-making Illustrate the role of ethical principles in guiding professional behaviour Justify the value of patient/person-centred practice Demonstrate engagement in reflective practice and continuing professional development
**Structure and Content**	Indicative syllabus/content Week 1: Legislation 1 Legislation 2 Lifelong-learning Week 2: Code of conduct Approaches to ethical decision-making Being an advocate Week 3: Being professional Industry Perspective on Case Regulatory Perspective on Case
**Learning Time**	Each week students will have a combination of learning online and in their placement setting. Students will spend 30 h per week learning in their placement, and this will be supported by 10 h of directed study (online learning activities, completing core and recommended reading, and completing assessment activities). Students will also be expected to complete self-directed learning activities of 10 h per week.
**Assessment**	Case-based Online Assessment [100%] Independent work (including critical appraisal and decision-making) [50%] Collaborative group decision making and peer review [50%] CA sign-off on Level 3 in relevant behaviours listed above by the end of the 4-month placement
**Indicative Reading**	**See ‘full’ module descriptor—Includes two core references**

**Table A3:** Case study development guidelines (First module delivered 2018).

**(a) Scenario/case development [18,20,21].**	Develop a scenario that raises various professional/ethical concepts. In order to create a dilemma, two concepts (at least) are in conflict i.e., an ethical/professional dilemma involves two or more action options, each of which is individually convincing, mutually exclusive and jointly demanding, and none of which is regarded as being fully aligned with all professional rules, codes, guidelines and ethical concepts. In order to assure the case study represents one or more dilemma(s), the case must avoid scenarios where one professional/ethical concept is ‘obviously’ dominant. Scenarios may incorporate more than one conflict of professional/ethical concepts. The scenario should not permit ‘escape’ e.g., to the letter of the law or to a standard clinical decision-making flowchart. As individual review of the scenario is not meant to be a challenging test of knowledge, footnotes are included to explain any terms or medicines relevant to the dilemma. The scenario/video finishes at a moment in time that can legitimately require an ‘immediate decision in less than ideal circumstances’.
**(b) Question posed at the end of the scenario (September 2018).**	The question aligns with the module LOs and content, and with the rubric provided to students. Questions are updated each year and may be prefaced with additional information related to the case study.
**(c) Development of 12 Action options for use in the first Module [18,20,21]**	12 action options are required for each case. In order to facilitate variation of options in subsequent years, Module coordinators are encouraged to prepare a minimum of 18 (6 × 3) when first developing a case. Development of the 12 options is not intended to be a ‘scientific’ process, rather that the action options are approximately equivalent and/or that none is obviously better than the other three and ordering of the 12 behaviours in the final ‘learning and assessment’ presentation is not random. Options are dispersed throughout (a) to (l), front loading from the ‘personal interest’ category, and ‘back-loading’ slightly from the ‘societal best interests’ options. Write 4–6 actions/behaviours that represent [behaviour in the person’s own interest] or [avoidance of taking responsibility] or [not advocating for an action that should be taken] for the situation (i.e., not defensible from a professionalism perspective); Write 4–6 actions/behaviours that represent behaviour focussed on maintaining rules/norms/codes e.g., that are articulated by legislature, policy documents or published professional standards i.e., behaviours that peers would debate whether ‘the behaviour was questionable or ‘defensible’ from a professionalism perspective. Write 4–6 actions/behaviours that represent behaviour in the patient’s and/or ‘society’s ‘best interests’ i.e., behaviour that peers would generally consider to be Highly Defensible’ from a professionalism perspective.

The components are a professional-specific scenario written as a script for recording of the video, a question posed to students to prompt critical review of the scenario and 12 action options that might be taken immediately after encountering the scenario.

The case aligns with the Module LOs and incorporates material covered in the module.

(a). Year 4 Professional Practice Module

Box A1 Case study—narration for videoclip. Narrator to introduce as follows: It is after 5 p.m. in a large teaching Children’s Hospital in Ireland and Dr. Claire Browne, Consultant Paediatrician in the hospital was hoping to catch someone in its pharmacy department before it closed. She has a drug query relating to a young child on the neurology ward that she would like to discuss and was hoping to catch Sean, the pharmacist on that ward before he left for the day. Dialogue: Claire: Hi Sean, I was down at the pharmacy hoping to catch you before you headed off. I see you’re ready to leave but any chance I could have a quick word? Sean: No problem Claire, but I need to pick up Killian from the crèche by six so it will have to be quick. Claire: Great Sean, are you familiar with Eve Brennan on the neurology ward with you is on IV sodium valproate because of her swallowing difficulties. Sean: Yes, I am. Claire: We need to get her onto another formulation as soon as possible. Anything by mouth is not an option. Sean: I understand that. Claire: I was talking to a colleague in my former hospital in London and he suggested sodium valproate suppositories. He mentioned a 300 mg strength suppository. Do you know anything about them? Sean: I’ve heard about them but never come across them in practice. There is no authorised sodium valproate suppository available in Ireland as far as I know so it won’t be on the hospital formulary. Claire: Does that mean we can’t use it? Sean: Not necessarily Claire: Even though I’m back from London six months, this whole exempt medicines situation here has me mightily confused. Sean (smiling): It’s not that bad really Claire (cheerfully): Isn’t it? Any chance you could investigate whether the suppositories are an option for Eve? Until, we get her off IV valproate and stabilised on another formulation, we can’t start planning for her discharge. Sean: I know can you leave it with me. It will probably be a day or two. Claire: Thanks Sean, that would be great. Sean: Fine so. I will need to have a look at her kardex to get more details but leave it with me. Claire: Perfect, let me know if you need to clarify anything Sean: Will do, bye now MPharm Year 4 PP module case development–video script 180524 ML/LS/CR Claire: Bye now, blame me if the crèche staff give out to you! Sean and Claire depart in opposite directions down the corridor. The camera follows Sean who thinks aloud as follows: This is an interesting one. We need to get Eve stabilised on another valproate formulation so that the team can try and get her home. I haven’t come across these suppositories before. There’s lots of issues to be investigated. It took a while to get her seizures controlled so looking at an alternative drug isn’t an option. “I wonder if sodium valproate suppositories are authorised in any other EU country or will it be a “specials” order Sometimes, these exempt medicines can be very expensive so that is something else to be considered. If Eve is discharged back home on these suppositories, then we will need to investigate whether she will be able to get them dispensed under one of the drug reimbursement schemes from her local pharmacy. And that just for starters……busy day ahead tomorrow! Narrator says? … If you were Sean, what would you do next?

(b) First Module Professional Practice Case Study Video Question Posed

Identify the main professional and/or ethical concepts, principles in the Code of Conduct and/or relevant legislation that you think might be at risk in this scenario, list what stakeholders you think need to be contacted, and then recommend, with justifications, what the pharmacist should do next i.e., directly after this scenario occurs (wordcount limit 250 words).

(c) Action options that students were required to rate and rank online in 2018

**Table A4:** Professional practice module (individual work) rubric.

Year 4: Professional Practice Module 190228v2.0 CR/LS/ML	Rubric for Individual Component: Individual Constructivism, Critical and Integrative Thinking and Reflective Practice are Emphasised.						Total: 50% of Module MARKS
Criteria (Weighting is 10% per Criterion)	Exceptional Level 5 × 5	Excellent Level 4 × 5	Very Good Level 3 × 5	Borderline Level 2 × 5	Limited Level 1 × 5	Unacceptable Level 0 × 5	Aligned with Learning outcome Numbers
Identifies Professional/Ethical concepts in the scenario and what leads to a dilemma, and critical review in a professional manner.	Answer fulfils all requirements for a level 4 answer and, in addition, is exceptional in its overall arguments and presentation	Comprehensive and accurate coverage of the concepts in the scenario, and the dilemma itself and clear linkage with values in the CoC, frameworks for decision-making, relevant legislation and issues of consent and confidentiality as appropriate.	Accurate and well informed regarding concepts in the scenario and the dilemma itself and links with CoC or frameworks for decision-making with some omissions or errors.	Generally accurate with respect to identification of concepts with some omissions or errors. Poor linkage with CoC, frameworks for decision-making or legislation as appropriate. Or posts to group’s forum <24 h ‘late’	Does not directly address the concepts, the dilemma or link with CoC, frameworks for decision-making or legislation as appropriate. Or posts to group’s forum >24 h ‘late’	Does not address the concepts in the dilemma. Or does not answer the question(s) posed. Or does not post to group’s forum.	1, 2, 3, 4, 5, 6, 7, 8
Makes and justifies decisions in a manner that reflects the statutory Code of Conduct for pharmacists and pharmacy and medicines law. (CCF)	Answer fulfils all requirements for a level 4 answer and, in addition, is exceptional in its overall arguments and presentation	Answers the question, offers a critical analysis of the scenario and justifies action choice in an integrated, logical, and relevant manner. Wordcount: 230–270	Answers the question, offers some analysis of the scenario and justifies action choice in a relevant manner. Wordcount: 230–270	Answers the question and offers some analysis of the scenario without specifically justifying the choice made. WC: <230 or >270	Answers the question(s), relating answers to questions posed, but states own opinions and choices rather than seeking to explain a reasoned action option. WC: <200 or > 300	No evidence of trying to develop a reasoned approach to choosing and justifying an action option.	1, 2, 3, 4, 5, 6, 7, 8
Action choices aligned with ‘expert’ view. (i.e., student rates and ranks action options provided).	Completes activity and posts to group’s forum by the deadline(s) and both top rank choices align with expert view.	This grade is not an option for this criterion	Completes activity and posts to group’s forum by the deadline(s) and one top rank choice aligns with expert view.	Completes & posts to group’s forum within 24 h of deadline, and one top rank choice aligns with expert view.	Completes & posts to group’s forum more than 24 h after deadline, and neither top rank choice aligns with expert view.	Does not complete or does not post to group’s forum.	4, 7
Take your choice of least preferred option and explain how a pharmacist might justify this choice as a preferred course of action. (100 words)	Answer fulfils all requirements for a level 4 answer and, in addition, is exceptional in its overall arguments and presentation.	Demonstrates understanding of how poor professional decision-making might arise and how pharmacists might try to justify same. Wordcount: 90–110.	Demonstrates understanding of how poor professional decision-making might arise or how pharmacists might try to justify same. Wordcount: 90–110.	States examples of alternate decisions that might be taken without specifying how pharmacists might try to justify same. Or posts to group’s forum <24 h ‘late’. WC: <90 or >110	Gives one example of an alternate decision that might be taken but does not clarify how a pharmacist might try to justify same. Or posts to group’s forum >24 h ‘late’. WC: <80 or >120	Examples of alternate actions /justifications are not plausible in the context of pharmacy practice. Or does not post to group’s Forum.	2, 5, 6
Reflects on own initial response to the scenario in the context of the 12 action options provided plus general reflection in the intervening two days. Refer to Learning Unit 3, Lifelong Learning. 150 words	Answer fulfils all requirements for a level 4 answer and, in addition, is exceptional in its overall arguments and presentation	Critical reflection: This form of reflection shows, in addition to dialogic reflection, evidence that the learner is aware that the same actions and events may be seen in different contexts, and that the different contexts may be associated with different explanations. Wordcount: 135–165	Dialogic reflection: This writing suggests that there is a ‘stepping back’ from the events and actions which leads to a different level of discourse. There is a sense of discourse with the ‘self’ and an exploration of the role of the ‘self’ in events and actions. The quality of judgements and of possible alternatives for explaining and hypothesising are also considered. The reflection is analytical or integrative, linking factors and perspectives. Wordcount: 135–165	Descriptive reflection: This is a description of events, that also shows some evidence of deeper consideration … but in relatively descriptive language. There is no real evidence of the notion of alternative viewpoints in use. Or posts to group’s forum <24 h ‘late’. WC: <135 or >165	Descriptive writing: This is a description of events …. It does not show evidence of reflection. Note: Some parts of a reflective account will need to describe the context—but in the case of ‘descriptive writing’, the writing does not go beyond description. Or posts to group’s forum >24 h ‘late’ WC: <120 or >180	Does not complete the reflection. Or does not post to group’s forum.	1, 3, 8

**Table A5:** Professional practice module (group work) rubric.

Year 4, Professional Practice Module 190229v2.0 CR/LS/ML	Rubric for Group/Teamwork Component (accounts for 50% of marks); Emphasis on Social Constructivism, Professionalism, and Peer Review						Total 50% of Module Marks
Criteria (Weighting is 10% per criterion)	Exceptional Level 5 × 5	Excellent Level 4 × 5	Very Good Level 3 × 5	Borderline Level 2 × 5	Limited Level 1 × 5	Unacceptable Level 0 × 5	Aligned with Learning Outcomes
Strategy to address the scenario posted to include: Content, appropriateness of advice, structure and referencing, Note 1: Referencing is to be Vancouver style. References are not included in ‘wordcount’ calculation. Note 2: For this criterion, the same mark will be awarded to all group members.	Answer fulfils all requirements for a level 4 answer and, in addition, is exceptional in its overall arguments and presentation.	Comprehensive, accurate, and well-informed overage of the concepts in the scenario/dilemma. The group provides cogent, well-reasoned ‘advice’, derived from the evidence base, to the pharmacist. References are of a high standard and are well integrated with the advice (Vancouver style). Wordcount (WC): 450–550 words	Accurate and well informed regarding concepts in the dilemma. The group posts appropriate ‘advice’ to the pharmacist References are of a high standard but not integrated with the argument (Vancouver style). WC: 450–550	Occasional omission of key factors that should be addressed in response to the scenario presented. Advice provided meets minimal standard. References are provided, but are of a minimal standard. Advice posted after the deadline, but within the same day. WC: <450 or >550	Omission of many of the key factors that should be addressed in response to the scenario presented. Or advice provided is not of minimal standard. Or referencing is absent or of a very poor standard. Or advice is posted after the due date, but up to 24 h after due date. WC: <400 or >600	Advice has not been posted within 24 h after due date. Or advice failed to fulfil any of the module learning outcomes.	1, 2, 3, 7, 8
Demonstrates professionalism and observes netiquette when preparing 500 words of advice, and when ranking action options as a group.	Answer fulfils all requirements for a level 4 answer and, in addition, is exceptional in its overall arguments and presentation.	The group at all times engaged in the consideration of the scenario in a highly professional, patient-focused, and dignified manner.	The group generally engaged in the consideration of the scenario in a mostly professional, patient-focused, and dignified manner.	The group intermittently engaged in the consideration of the scenario in a professional, patient-focused, and dignified manner.	Significant breach of netiquette on an individual or collective basis which is recognised, but not satisfactorily addressed within the group discussion.	Significant breach of netiquette on an individual or collective basis which does not appear to have been recognised. Or not submitted.	1, 2, 3, 8
Achieves reasoned consensus regarding most and least preferred action options in order of preference, using a clearly defined process.	Answer fulfils all requirements for a level 4 answer and, in addition, is exceptional in its overall arguments and presentation.	Achieves reasoned consensus regarding most and least preferred actions (3 of each), using a clearly defined process.	Achieves reasoned consensus regarding most and least preferred actions (3 of each) with tendency to use ‘voting’ to reach decision(s) (as opposed to using voting to inform decision-making process.	Achieves reasoned consensus regarding most and least preferred actions (3 of each) without clearly identifying ranking.	Achieves consensus regarding most and least preferred action options in order of preference. Any individual student contributions are minimal and are independent of group discussion and do not demonstrate reflective listening.	Group does not post all 6 choices by the due date Any individual student failing to make 3 contributions to the discussion, to the minimum standard required.	1, 3, 4, 6, 7, 8
Group undertakes and agrees Peer Review A in a manner that demonstrates professionalism and observes netiquette Note that: Learning Unit 7, ‘Being Professional’, includes guidance on peer review. This peer review activity should reflect expectations outlined in Criterion 1 of this rubric.	Answer fulfils all requirements for a level 4 answer and, in addition, is exceptional in its overall arguments and presentation.	Provides a specific, targeted, realistic, implementable sentence of ‘reinforcing’ feedback. Provides a specific, targeted, realistic, implementable sentence of ‘how advice might be improved’. The group at all times engaged in the peer review process in a highly professional, patient-focused, and dignified manner.	Provides an appropriate sentence of ‘reinforcing’ feedback. Provides an appropriate sentence of ‘how advice might be improved’. The group generally engaged in the peer review process in a mostly professional, patient focused and dignified manner.	Provides a specific, but non-implementable sentence of ‘reinforcing’ feedback. Or provides a non-specific sentence of ‘how advice might be improved’. The group intermittently engaged in the peer review process in a professional, patient-focused, and dignified manner.	Provides feedback that is not specific, is unrealistic and is non-implementable. Or significant breach of netiquette on an individual or collective basis which is recognised, but not satisfactorily addressed within the group discussion.	Doesn’t provide feedback. Or significant breach of netiquette on an individual or collective basis which does not appear to have been recognised.	1, 3, 4, 5, 8
Group undertakes and agrees Peer Review B in a manner that demonstrates professionalism and observes netiquette. Note that: Learning Unit 7, ‘Being Professional’, includes guidance on peer review. This peer review activity should reflect expectations outlined in Criterion 1 of this rubric.	Answer fulfils all requirements for a level 4 answer and, in addition, is exceptional in its overall arguments and presentation.	Provides a specific, targeted, realistic, implementable sentence of ‘reinforcing’ feedback. Provides a specific, targeted, realistic, implementable sentence of ‘how advice might be improved’. The group at all times engaged in the peer review process in a highly professional, patient-focused, and dignified manner.	Provides an appropriate sentence of ‘reinforcing’ feedback. Provides an appropriate sentence of ‘how advice might be improved’. The group generally engaged in the peer review process in a mostly professional, patient focused, and dignified manner.	Provides a specific, but non-implementable sentence of ‘reinforcing’ feedback. Or provides a non-specific sentence of ‘how advice might be improved’. The group intermittently engaged in the peer review process in a professional, patient-focused, and dignified manner.	Provides feedback that is not specific, is unrealistic and is non-implementable. Or significant breach of netiquette on an individual or collective basis which is recognised, but not satisfactorily addressed within the group discussion.	Doesn’t provide feedback. Or significant breach of netiquette on an individual or collective basis which does not appear to have been recognised.	1, 3, 4, 5, 8

**Table A6:** Summary of student learning and assessment activities (first module).

Week	Learning and assessment activity/-ies
OW	Student access to all VLE functionality required for activities and assessment are confirmed during OW including: System of weekly announcements is introduced. MC discussion Forum introduced and clarifies that the MC will respond to queries at least twice weekly. Three LUs introduce students to various aspects of placement learning. A video demonstration of activities/submissions completion on the VLE. Concept of core references is introduced (online module student guidance booklet and the APPEL handbook). Video of a ‘placement dilemma’ is available for download at 1 p.m. on Wednesday afternoon. Student rates and ranks 12 action options related to the video. Peer learning by means of contributing responses to prompt questions and commenting on peer contributions on four Discussion Forums aligned with core references.
Moduleweek 1	Online activities in week 1 are formative—i.e., no academic credit is awarded. Two core references are available to students online. Three LUs are released to students. Peer learning is initiated—i.e., students respond to prompt questions and comment on peer contributions on Forums aligned with core references.
Moduleweek 2	Three LUs are released to students. Wednesday 1:00 p.m.: Students are presented with a video encompassing a professional dilemma, and a question to consider – and must prepare and submit online a response of 250 words. Once 250-word response has been submitted, the 12 ‘Action options’ are provided and students submit individual rating and ranking online. Students consider their choice of least preferred option and prepare and submit a (100 word) explanation of how a pharmacist might justify this choice as a preferred course of action. By Friday 9:00 p.m.: Students reflect on their own initial response to the scenario in the context of the 12 action options plus general reflection in the intervening two days to prepare and submit a reflection (150 words).
Module week 3	The final three LUs for the module are released to students. Group allocations are visible to students i.e., they can ‘see’ names of ‘their own’ group members. By Monday night, each group member posts his/her four individual assignments to the group’s forum i.e., 250 word answer to the scenario, choices related to ranking of action options, 100 word explanation of the reasoning a pharmacist might use to justify the action option the student listed as the least preferred option, and the 150 word individual ‘reflection’. Groups have until Wednesday of week 4 to complete discussions.
Module week 4	Groups must agree ranking of three most and least preferred action options, and prepare 500 words of advice to the pharmacist, intern, patient, other stakeholder in the case study and post both as final contribution to the group’s forum by 5:00 p.m. on Wednesday week 4.
Module week 5	Groups are provided with 500 words of advice written by two other groups. Groups must agree two sentences of feedback for each advice one sentence ‘to put things right, and the other reinforcing what is ‘good’ [39]. Group tasks must be submitted as the last post on the relevant forum by 5:00 p.m. on Wednesday week 5.
